# Erythropoietic protoporphyria

**DOI:** 10.1186/1750-1172-4-19

**Published:** 2009-09-10

**Authors:** Mario Lecha, Hervé Puy, Jean-Charles Deybach

**Affiliations:** 1 Department of Dermatology, Hospital Clinic, University of Barcelona, Barcelona, Spain; 2 AP-HP, Centre Français des Porphyries, Hôpital Louis Mourier, 178 rue des Renouillers, 92701 Colombes CEDEX, France; 3 INSERM Unité 773, Centre de Recherche Biomedicale Bichat-Beaujon, Université Paris Diderot, site Bichat, 75018, Paris, France

## Abstract

Erythropoietic protoporphyria (EPP) is an inherited disorder of the haem metabolic pathway characterised by accumulation of protoporphyrin in blood, erythrocytes and tissues, and cutaneous manifestations of photosensitivity. EPP has been reported worldwide, with prevalence between 1:75,000 and 1:200,000. It usually manifests in early infancy upon the first sun exposures. EPP is characterised by cutaneous manifestations of acute painful photosensitivity with erythema and oedema, sometimes with petechiae, together with stinging and burning sensations upon exposure to sunlight, without blisters. These episodes have a variable severity depending on the exposure duration and may result in chronic permanent lesions on exposed skin. As protoporphyrin is a lipophilic molecule that is excreted by the liver, EPP patients are at risk of cholelithiasis with obstructive episodes, and chronic liver disease that might evolve to rapid acute liver failure. In most patients, EPP results from a partial deficiency of the last enzyme of the haem biosynthetic pathway, ferrochelatase, EC 4.99.1.1/FECH (encoded by the *FECH *gene). EPP appears to be inherited as an autosomal dominant disease, the clinical expression of which is modulated by the presence of the hypomorphic *FECH *IVS3-48C allele *trans*, but recessive inheritance with two mutated *FECH *alleles has also been described. In about 2% of patients, overt disease was recently shown to be caused by gain-of-function mutations in the erythroid-specific aminolevulinic acid synthase 2 (*ALAS2*/ALAS, EC 2.3.1.27) gene and named X-linked dominant protoporphyria. Diagnosis is established by finding increased levels of protoporphyrin in plasma and red blood cells, and detection of a plasma fluorescence peak at 634 nm. Investigations for hepatic involvement, ferrochelatase activity level, genetic analysis (*FECH *mutations, presence of the hypomorphic *FECH *IVS3-48C allele *trans *and *ALAS2 *mutations) and family studies are advisable. Differential diagnosis includes phototoxic drug reactions, hydroa vacciniforme, solar urticaria, contact dermatitis, angio-oedema and, in some cases, other types of porphyria. Management includes avoidance of exposure to light, reduction of protoporphyrin levels and prevention of progression of possible liver disease to liver failure. As the major risk in EPP patients is liver disease, a regular follow-up of hepatic involvement is essential. Sequential hepatic and bone marrow transplantation should be considered as a suitable treatment for most severe cases of EPP with hepatic involvement. EPP is a lifelong disorder whose prognosis depends on the evolution of the hepatic disease. However, photosensitivity may have a significant impact on quality of life of EPP patients.

## Disease names and synonyms

Erythropoietic protoporphyria, Protoporphyria, Haem synthetase deficiency, Ferrochelatase deficiency, X-linked dominant protoporphyria (XLDPP), Erythrohepatic protoporphyria (no longer used).

## Definition

Erythropoietic protoporphyria (EPP) is a painful photodermatosis without blisters caused by inborn errors of the haem biosynthetic pathway due, in the majority of patients, to a deficient activity of the enzyme ferrochelatase (ferrohaemprotolyase, haem synthetase, ferrohaem-protolyase, EC4.99.1.1, FECH (EPP), (MIM 177000) or caused, in about 2% of patients, by a gain of activity of the erythroid specific aminolevulinic acid synthase 2 (ALAS; EC 2.3.1.27). The latter results in a new protoporphyria named X-linked dominant protoporphyria (XLDPP), (MIM 300752) [1-3].

**Figure 1:**
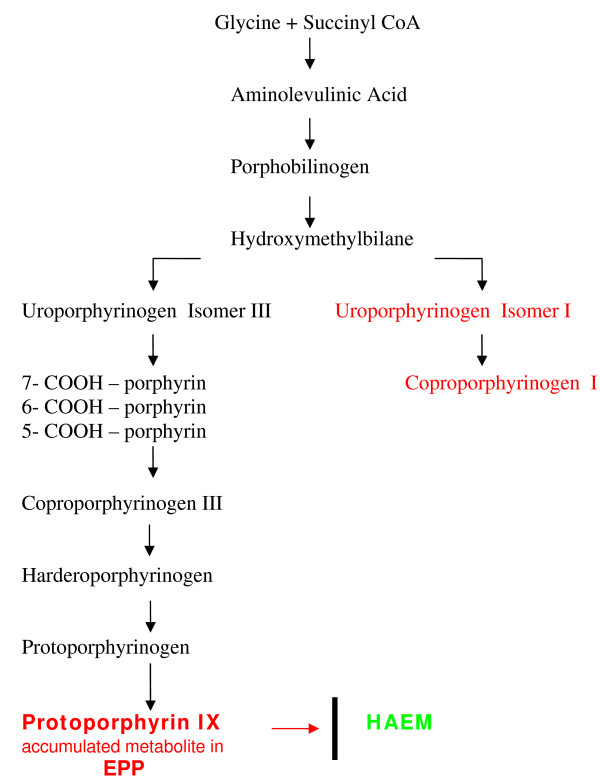
**The haem biosynthesis pathway**.

ALAS is the first enzyme of the haem synthetic pathway, which is synthesised by two different genes: *ALAS1 *in the liver and other tissues located on chromosome 3, and *ALAS2 *in the erythroid tissue located on chromosome X. The role of ALAS is to combine glycine and succinyl-coenzyme A to form delta aminolevulinic acid (ALA), the first substrate of the pathway in the mitochondrial matrix. FECH is the last of the eight enzymes acting sequentially in the haem biosynthetic pathway and is encoded by *FECH *gene on chromosome 18. The role of FECH is to catalyse the insertion of iron into protoporphyrin ring to generate the final product, haem (Fig 1).

Porphyrias may manifest in two clinical types: cutaneous photosensitivity (cutaneous porphyrias) or acute neuro-visceral symptoms (acute porphyrias). EPP belongs to the group of cutaneous porphyrias. In EPP, the enzyme deficiency causes accumulation of protoporphyrin in various tissues (skin, liver) and blood (erythrocytes and plasma). Skin protoporphyrin accumulation results in acute episodes of painful photosensitivity without blisters, which is the characteristic clinical manifestation of the disease.

The first comprehensive description of the disease was published by Magnus *et al*. in 1961 [1].

## Epidemiology

EPP has been described in patients worldwide. There are prevalence studies in different populations, with prevalence figures ranging between 1:75,000 (The Netherlands) and 1:200,000 (Wales) [4,5]. It seems that males and females are equally affected, as suggested by Holme *et al*. [6].

## Clinical description

### Skin symptoms: photosensitivity

**Figure 2:**
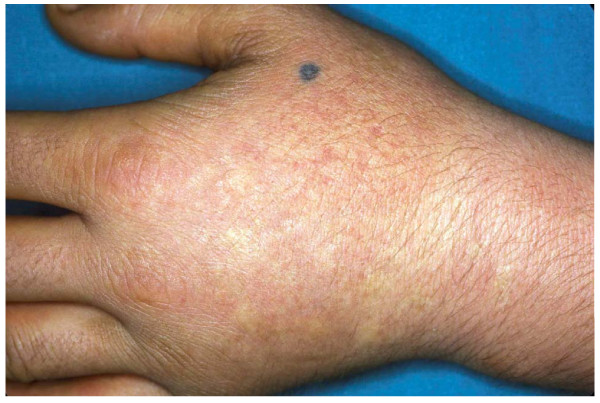
**Acute photosensitivity reaction in EPP**.

**Figure 3:**
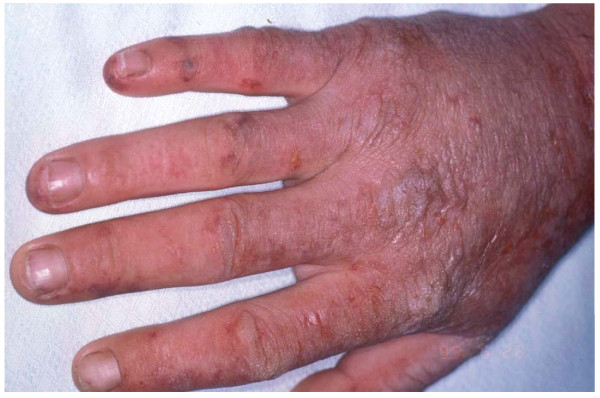
**Acute photosensitivity reaction in EPP**.

EPP manifests clinically by skin symptoms of immediate painful photosensitivity [2,7]. This manifestation starts usually in early infancy or childhood, upon the first sun exposures. Skin stinging, prickling and burning on exposed areas are the initial symptoms experienced by the patient during or shortly after sun exposure. These symptoms may have a variable duration, depending on the intensity and duration of sun exposure. If the exposure has been long enough, erythema and oedema may appear, together with petechiae in some cases. Areas affected are the face and dorsa of the hands, but photosensitivity reactions can also appear on any exposed skin area. Reactions may persist for hours up to several days. The appearance of vesicles or bullous lesions characteristic of other forms of cutaneous porphyria is unusual but may occur (Fig. 2, 3). Nail lesions (photoonycholysis and transversal leuconycholysis) are possible associated manifestations.

Late-onset cases have been reported but are exceptional and related to haematologic malignancies, although cases without this association have been described [8-10].

**Figure 4:**
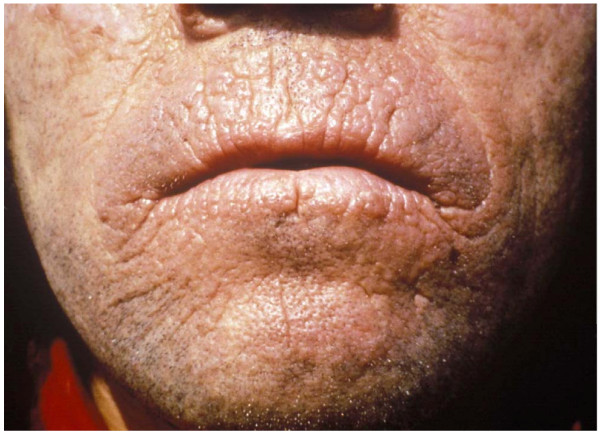
**Chronic skin lesions of EPP**.

Repeated photosensitivity episodes result in altered skin appearance with permanent changes, such as skin thickening with a waxy or leathery appearance, and areas of hyperkeratosis. These lesions are usually located on the dorsa of the hands and face, as these are skin areas usually exposed (Fig. 4). The lips show linear furrows and pseudo-rhagades that arte also characteristic. In contrast to other forms of cutaneous porphyria, lesions of milia, hypertrichosis or hyperpigmentation do not appear. This last cutaneous sign may only appear in patients with severe hepatic involvement presenting with cholestasis, itching and jaundice [2].

Seasonal palmar keratoderma has been reported in some EPP patients and in addition this may indicate autosomal recessive inheritance of the disease [11].

### Liver involvement

In EPP, the degree of severity of the hepatic manifestations is variable. Hepatic disease in EPP may include: cholelithiasis with possible obstructive episodes and chronic liver disease evolving to rapid acute hepatic failure [12,13].

The incidence of cholelithiasis is frequent in EPP (about 20% of patients). Gallstones with high protoporphyrin content are generated due to the accumulation of insoluble protoporphyrin and increased biliary protoporphyrin concentration [7].

Liver disease develops in association with EPP in 1-4% of cases [5,6], with usual features of visceral enlargement and portal hypertension. Liver disease in protoporphyria is related to the excess protoporphyrin cleared by the entero-hepatic circulation leading to paracrystalin protoporphyrin deposition in hepatocytes and precipitation in the biliary canaliculiy. The percentage of patients who will develop liver disease is not established, nor specific factors that may influence its development. It is not possible to predict whether or not acute hepatic failure will occur. Studies have revealed that an increase in coproporphyrin urinary excretion, together with a change in isomer predominance from isomer III to isomer I, and increasing levels of protoporphyrinaemia may precede this complication [14].

Progression of protoporphyric hepatic deterioration leads to splenomegaly, splenic sequestration of erythrocytes with haemolysis (which increases erythropoiesis) and protoporphyrin generation ending in fulminant hepatic failure [13-15].

Patients who have *FECH *mutations on both alleles or a gain of function mutation of *ALAS2 *[3] have an increased risk of liver disease though together they account for only a small proportion of those with liver disease.

An unsual neurological syndrome has been described with progressive polyneuropathy, swallowing difficulties and respiratory distress in several patients with end-stage liver disease due to protoprophyria [16]. Oral mucosa or eye lesions in EPP patents have recently been reported for the first time by Tsuboi H *et al*. [17].

### Pathological features of EPP

**Figure 5:**
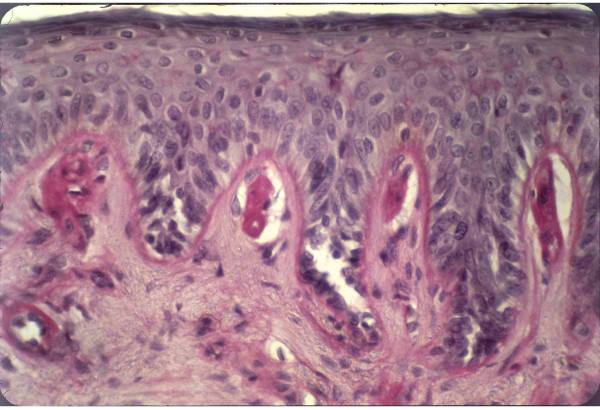
**Cutaneous histological features of EPP with PAS positive material deposition around papilar blood-vessels and dermo-epidermal junction**.

Cutaneous lesions result from the presence of protoporphyrin in erythrocytes and plasma in skin vasculature. Histologically, hyaline PAS-positive perivascular material deposits can be found together with extensive presence of fine fibrillar material, and reduplication of blood vessel walls and epidermal basal lamina. Although less prominent, these findings appear also in other cutaneous porphyrias (Fig 5).

Deposition of protoporphyrin in hepatocytes and in thrombi within the biliary canaliculi can be observed in the hepatic tissue. Characteristic pigment depositions are produced and under polarised light birefringent crystalline structures appear with the characteristic Maltese cross shape. Canaliculi may be dilatated and amorphous deposits within the lumen may be present [7].

### Biochemical and haematological alterations

Partial deficiency of FECH produces accumulation of free protoporphyrin IX. It can be detected in circulating red blood cells, in reticulocytes and in bone marrow erythroblasts. Red blood cells are therefore fluorescent under Wood's light. Protoporphyrin may also be detected in patients' plasma, that may also be fluorescent. Protoporphyrin is often excreted in stool and can be detected, but not in all patients. Urine is usually normal, except in cases with cholestasis and protoporphyrin hepatopathy. In this situation, an excess of urinary coproporphyrin isomer I can be demonstrated. FECH deficiency can be detected in different tissues with levels usually under 50% of normal (between 10 - 30%) to induce clinical manifestations [6].

Recently, families have been described in which EPP is inherited in an X-linked dominant pattern [3]. Patients with this disorder have normal FECH activities but higher erythrocyte total protoporphyrin concentrations than other types of EPP of which around 40% is zinc-protoporphyrin. This high proportion of zinc-protoporphyrin suggests that protoporphyrin accumulates because supply of both its metal substrates, Fe^2+ ^and Zn^2+^, becomes rate-limiting. Additionally, the increase in erythrocyte zinc-protoporphyrin in combination with a marked increase in free protoporphyrin appears to be a distinguishing feature of this form of EPP [18,19].

Microcytic anaemia occurs in 20% to 60% of patients. Erythropoiesis was impaired in most patients with dominant EPP from UK and France [18,19]. All had a downward shift in haemoglobin (Hb), women were slightly more anaemic than men. Iron stores, assessed by serum ferritin (sFn), were decreased by two-thirds, but normal serum soluble transferrin receptor-1 and iron concentrations suggested that erythropoiesis was not limited by iron supply. FECH deficiency in EPP appears to lead to a steady state in which decreased erythropoiesis is matched by reduced iron absorption and supply. This response may in part be mediated by protoporphyrin [18].

## Aetiopathogenesis

EPP clinical manifestations are consequence of protoporphyrin accumulation in various tissues. The protoporphyrin molecule absorbs light radiation in a range of wavelengths from 320 to 595 nm. The absorption of these wavelengths increases the energy content of the protoporphyrin molecule (inducing a triplet state) and enables the excess energy to be transferred to oxygen, resulting in a reactive oxygen species that may interact with many biological molecules, such as proteins, lipids and DNA (photodynamic reactions) [20].

As protoporhyrin is a hydrophobic molecule, it tends to accumulate in cellular membranes. Upon irradiation with the specific wavelengths mentioned above, a photodynamic reaction takes place in tissues where protoporphyrin is present (skin, red blood cells in skin blood vessels) and cellular membranes are damaged because of membrane lipids peroxidation. Oxygen species generation may also injure tissues by complement activation and mast cell degranulation phenomena that explain the vasodilatation and oedema components of the skin photosensitivity reactions in EPP patients.

Histological examination of the skin upon sun exposure in EPP patients reveals the presence of inflammatory reaction arround dermal capillaries, with inflammatory infiltration. Subsequently, a repair reaction ensues with collagen synthesis. This process is produced repeatedly and manifests histologically by a deposition of PAS-positive material around skin blood vessels, which, on the other hand, show reduplication of capillary basal membranes [21].

Protoporphyrin is highly toxic, independently of the photosensitized reactions. Possible mechanisms underlying this hepatic toxicity have been described, but none of these hypotheses have been confirmed. The basic consequence is that the biliary system is exposed to high concentrations of protoporphyrin that saturate the excretion capacity. Deposits of protoporphyrin are formed and further reduce excretion capacity. Hepatic inflammation may appear with disordered tissue regeneration, hepatic fibrosis and cirrhosis associated with biliary disease [7,21,22].

### Genetics

With only rare exceptions, EPP is an inherited disorder. Even after the identification of mutations in the *FECH *gene in EPP, the precise pattern of inheritance remained uncertain. The difference in FECH activity between clinically overt and latent individuals could not readily be explained by the prevailing view that inheritance was autosomal dominant with low clinical penetrance but was consistent with a three allele system. Recent genetic studies revealed different patterns of inheritance in EPP [23,24].

In most patients with EPP, a *FECH *mutation that markedly decreases or abolishes enzyme activity can be identified on only one allele [23,25-30]. The discovery that clinical expression of this type of EPP normally required a hypomorphic FECH IVS3-48C allele *trans *to the mutation was demonstrated in France [26,31] and was independently confirmed by studies from Japan, North America, Sweden, Israel, South Africa, the United Kingdom [28,30-34].

The pattern of inheritance of photosensitivity has been well documented by Went and Klasen [4] who investigated 91 families from the Netherlands, most of whom were likely to have had this type of EPP. About half the cases were sporadic. When a family contained more than one patient, affected siblings were more common than parent to child transmission. The number of families with patients in more than one generation increases with the population prevalence, occurring in 15% of French and 51% of Japanese families. In contrast, EPP caused by FECH deficiency has not been reported in black South African families [26,33].

*FECH *mutations on both alleles are an uncommon cause of EPP. To date, twenty-one symptomatic patients from 17 families have been reported. In the UK and France, this type of EPP accounts for about 4% of EPP families. Reported FECH activities, either measured in lymphocytes or estimated from *in vitro *expression studies, are within the median range of 9%. Molecular analyses show that most of the patients are compound heterozygotes. In marked contrast to the type of EPP described above, missense mutations make up 85% of the total and null mutations are unusual [28,30-34].

The pattern of inheritance of photosensitivity in these families is characteristic of an autosomal recessive disorder. There is an increased incidence of consanguinity, only siblings are affected and both parents are clinically normal. This last feature may in part be explained by the chance absence of a hypomorphic allele *trans *to the mutation [35,36],

Acquired somatic FECH mutations have been identified in a small number of patients in whom EPP has developed after the age of 40 years in association with myelodysplasia or myeloproliferative disorder [10,37].

Patients in which EPP is inherited in an X-linked dominant pattern have normal FECH activities but higher erythrocyte total protoporphyrin concentrations than other types of EPP of which around 40% is zinc-protoporphyrin. Two frameshift mutations have been identified in 8 families that lead to predicted disruption or deletion of the 19-20 C-terminal amino-acids of ALAS2. Prokaryotic expression studies show that both mutations markedly increase ALAS2 activity. Thus the C-terminal region of ALAS2 appears to inhibit enzyme activity; its removal by mutation leads to gain of function whereas all other previously described mutations in *ALAS2 *decrease activity and cause hereditary sideroblastic anaemia.

Despite advances in techniques for mutation detection and the discovery of gain in function mutations of *ALAS2 *as a cause of EPP, in about 5% of EPP families no mutations in neither *FECH *or *ALAS2 *genes have been identified. Disease in these families is strongly associated with inheritance of the hypomorphic *FECH *IVS3-48C allele and decreased FECH activity which suggests that most may have mutations in regions of the *FECH *gene that are not included in current strategies for mutation detection.

### Genotype/phenotype correlations

The cutaneous features of EPP, though varying in severity are remarkably uniform. No correlation between indices of severity of photosensitivity, such as age of onset or duration of symptoms, and genotype have yet been reported apart from the suggestion that the relatively common missense mutation, P334L, may cause only mild disease Apart from this instance, no correlation between erythrocyte protoporphyrin concentration and type of *FECH *mutation has been reported. Seasonal palmar keratoderma has to date been reported only in patients who are compound heterozygotes or homozygotes for *FECH *mutations [19,38].

A mainly literature-based study of 112 patients with a single *FECH *mutation, of whom 18 had severe liver disease, found that all patients with liver disease had a 'null' mutation (splicing defect, nonsense or frameshift) while none of the 20 patients with a missense mutation had liver disease; this difference was statistically significant [39]. Under-representation of missense mutations was also noted in a group of 15 patients with liver disease, all studied in the same laboratory, but comparison with a group without liver disease did not reach statistical significance [4,35]. Together these studies suggest that missense mutations that preserve significant amounts of residual activity may carry a lower risk of liver disease than 'null' mutations, a group that includes those misssense mutations that abolish FECH activity. However, 'null' mutations associated with liver disease are found more frequently in patients without liver disease; an observation that indicates the probable importance of genetic factors outside the *FECH *locus and acquired factors in the pathogenesis of protoporphyric liver disease [40].

## Diagnosis

**Figure 6:**
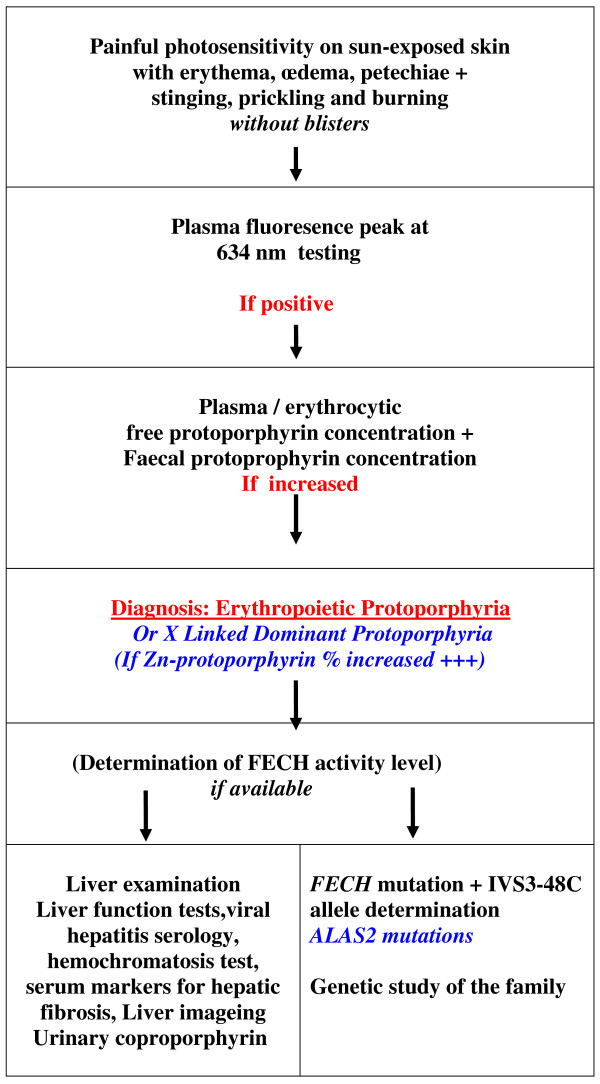
**EPP diagnosis**.

Photosensitivity, with the characteristic painful inflammatory reaction without blistering, is the clinical basis for diagnosis of EPP. Screening by fluorescence microscopy for the presence of fluorescent erythrocytes - fluorocytes - in a fresh unstained blood smear may be the first step in confirmation of the EPP diagnosis. This step should be followed by the detection of a plasmatic fluorescence peak at 634 nm (630 - 635) and the demonstration of high free protoporphyrin levels in plasma and erythrocytes or a higher content and percentage of zinc-protoporphyrin in XLDPP patients. Presence of increased protoporphyrin in the stools may also be a useful diagnostic finding. These data confirm the diagnosis of EPP (Fig 6). In addition, the levels of FECH activity in selected tissues may be determined.

Hepatic function should be tested and abdominal ultrasonographic examination should be performed to detect cholelithiasis.

Screening for either *FECH *gene mutations and for the presence of the *IVS3-48C *hypomorphic *FECH *allele or *ALAS2 *gain of function mutations, together with studies of family members, may also be proposed. These data will be useful for genetic counselling [7].

## Differential diagnosis

Clinically, EPP should be differentiated from phototoxic drug reactions, hydroa vacciniforme, solar urticaria. Contact dermatitis and angioedema or even other types of curaneous porphyria could also be considered. Chronic lesions should be differentiated from lipoid proteinosis [7].

Biochemically, excess protoporphyrin should be present in the form of free protoporphyrin. Elevated zinc chelated-protoporphyrin levels could indicate iron deficiency or lead poisoning but also now the X-linked EPP due to *ALAS2 *gain of function mutations. Elevated levels of zinc-protoprophyrin may be detected in homozygotes with other inherited forms of porphyria [7,41].

## Genetic counselling

In the classic EPP with *FECH *mutation on one allele and hypomorphic *FECH IVS3-48C *allele in *trans *position, the probability for the offspring of an EPP patient to present the disease is less than 1:40 (less than 2.5%). Hypomorphic *FECH *allele in the general population (Caucasians) has a prevalence of around 10% [26,40]. Screening for the presence of the *FECH IVS3-48C *hypomorphic allele in the EPP patient's partner allows more accurate estimation of this probability.

During pregnancy, EPP is usually not a problem neither for the mother nor for the baby. With the exception of one recently published case, the reported cases of pregnancy in patients with EPP have revealed that symptoms of photosensitivity improve during pregnancy, with reduction of erythrocyte protoprophyrin levels [42].

## Management

### Management of photosensitivity

Management of photosensitivity is an essential therapeutic measure. Avoidance of sun exposure, even through window glass (*e.g*. car driving), is the most practical way of preventing photosensitivity reactions in EPP patients. The use of adequate clothing (hats, glasses, gloves) and sunscreens is also advisable. Due to the fact that protection should cover both long-wave radiation plus visible light, physical sunscreens are more effective.

Management of photosensitivity reactions includes the use of: wet (cold) water compresses applied to the affected areas. Topical corticosteroids may be prescribed but are usually ineffective. Oral treatment, if required, may include non-steroidal anti-inflammatory drugs or oral corticosteroids.

Betacarotene has been used extensively, with the aim of improving light tolerance in EPP patients. Some benefit from its use has been experienced by patients. The following doses of betacarotene are recommended: up to 90-120 mg/day in children and up to 180-300 mg/day in adults. Recent studies, however, have suggested inverse associations between carotenoids and lung cancer mainly in smokers [43,44].

Other treatment approaches include the administration of Vitamin C and E, and cysteine (500 mg/twice daily), orally. Antihistamines may also be used as they may limit the phototoxic reaction.

Except for betacarotene, there is a lack of firm evidence proving the effectiveness of all these treatment approaches [45]. The duration of treatments directed to improve photosensitivity is related to the ratio risk/benefit obtained by patients individually. UVB/NBUVB phototherapy or PUVA may be used in order to induce tanning and improve the sunlight tolerance. The duration of the treatment is limited by the guidelines of phototherapy and depends also on the patient benefit obtained in reduction of photosensitivity [46].

Afamelanotide (an alpha-melanocyte-stimulating hormone analogue that induces epidermal melanin formation) has recently been shown to have beneficial effects in patients with EPP [47].

EPP patients may present photosensivity reactions during surgical procedures due to exposure to the theater lighting or other surgical light conducting devices in laparoscopy, endoscopy or dental procedures. In these cases, the application of yellow filters blocking the radiation under 460 nm offers the best protection in keeping with a level of normal acceptable visibility [48,49].

### Reduction of protoporphyrin levels

Regarding the reduction of excess protoporhyrn in patients with EPP, two approaches can be considered:

a) Reduction of erythropoiesis. This may be achieved by exchange transfusion or hypertransfusion. It is not clear if haematin may temporarily supress erythropoiesis by modifying the activity of the rate-limiting first enzyme of the haem synthetic pathway, ALA synthase. However, this approach cannot be considered as a long-term treatment of the disease [50-52].

b) Cholestyramine (a bile sequestering agent) may be used to increase the elimination of the excess protoporphyrin through the biliary system. It binds the excess protoprophrin, enhancing its faecal elimination, reducing its plasma and red blood cell concentration, and preventing the protoporphyric hepatopathy. The suggested dose of cholestyramine is 4-16 g daily. Other similar drugs have been considered, such as chenodeoxycholic acid and ursodeoxycholic acid [53-55].

However these bile sequestering agents might have no or even adverse effect in a mouse model of EPP with progressive liver disease and should be therefore used with caution (JC Deybach and H Puy, personal communication).

### Management of microcytic anaemia

Patients studied had a mild microcytic anaemia and thrombocytopenia, as shown by the downward shift of hematologic parameters, which positively correlated with the amount of erythrocyte PPIX. Interestingly, erythropoiesis did not seem to be limited by iron supply in patients, since serum iron and soluble transferrin (Tf) receptor (sTfR) were normal. Therefore oral iron supply is usually found ineffective in trying to correct the usual mild anaemia in EPP patients and is therefore not recommended. However intravenous iron supply or even blood transfusion should be considered in EPP with a more pronounced anaemia [7].

### Management of liver disease

Liver transplantation is the treatment for end-stage liver disease. It was used for the first time in an EPP patient in 1980 [55]. Progressive liver disease in EPP patients should be considered a medical emergency and, therefore, liver transplantation has ben indicated in patiens with EPP.

It is evident that liver transplantation does not alter the consequences of FECH deficiency and overproduction of proporhyrin. Therefore, the EPP patients continue to present with the typical symptoms and same risks. Bone marrow transplantation should be considered and, in fact, the ideal treatment should be sequential liver and bone marrow transplantation, which has already been performed with success [56,57].

As the experience with liver and bone marrow transplantations in EPP patients increases, rationale for patient selection, indication for transplantation and timing either for bone marrow, liver or sequential transplantations can be established. Knowledge concerning tolerance of the immunosuppressive therapy and long-term complications in EPP patients also needs to be gained. A proposal of guidelines to identify liver disease in EPP patients has been published by Anstey and Rift very recently [58]. Special attention should be devoted to EPP patients bearing *FECH *null mutations with recessive inherited disease, those belonging to families with more than one member with manifested disease or those with X linked protoporphyria.

Liver biopsy should be indicated in patients with null mutations or recessive inherited disease, patients from families with previous cases of EPP liver disease, and in individuals with EPP plus other risk factors for liver disease (such as hepatitis, haemochromatosis, alcoholic or non-alcoholic fatty liver disease, abnormal liver function tests). Special attention should also be given to patients with sudden worsening of photosensitivity, rising blood protoporphyrin levels, increasing urinary coproporphyrinuria isomer I and decreasing faecal protoprophyrin.

EPP patients should be monitored through a periodic non-invasive liver testing protocol that includes liver function tests, viral hepatitis serology, haemochromatosis tests and analysis for serum markers of hepatic fibrosis. Liver scanning for detection of gallstones, and computed tomography (CT) and magnetic resonance imaging (MRI) liver studies should also be included.

EPP patients should be vaccinated against hepatitis. They should avoid alcohol intake.

## Prognosis

EPP is a lifelong disease whose prognosis depends on the evolution of the hepatic disease that may lead to potentially fatal liver failure. Regarding life quality aspects, only one study has addressed this question in porphyric patients and, specifically, in EPP patients. It has been concluded that photosensitivity may significantly modify the life style of EPP patients, with a marked impact on the quality of life and with scores that appear higher in EPP patients as compared to those for other skin diseases considered as severe [6].

## Abbreviations

EPP: erythropoietic protoporphyria; FECH: ferrohaemprotolyase; ALAS: aminolevulinic acid synthase; XLDPP: X-linked dominant protoporphyria; ALA: aminolevulinic acid; Hb: haemoglobin; SFn: serum ferritin; Tf: transferrin; STfR: soluble transferrin receptor; CT: computing tomography; MRI: magnetic resonance imaging

## Competing interests

The authors declare that they have no competing interests.

## Authors' contributions

ML wrote the first draft of the manuscript; all authors revised the manuscript and approved the final version.
